# Computational Modeling of Properties of Quantum Dots and Nanostructures: From First Principles to Artificial Intelligence (A Review)

**DOI:** 10.3390/nano15040272

**Published:** 2025-02-11

**Authors:** Grzegorz Matyszczak, Krzysztof Krawczyk, Albert Yedzikhanau

**Affiliations:** Department of Chemical Technology, Faculty of Chemistry, Warsaw University of Technology, Noakowski Str. 3, 00-664 Warsaw, Poland; krzysztof.krawczyk@pw.edu.pl (K.K.); albert.yedzikhanau.stud@pw.edu.pl (A.Y.)

**Keywords:** nanostructures, quantum dots, mathematical modeling, computation, DFT, artificial intelligence, machine learning

## Abstract

Nanomaterials, including quantum dots, have gained more and more attention in the past few decades due to their extraordinary properties that make them useful for many applications, ranging from catalysis, energy generation and storage, biotechnology, and medicine to quantum informatics. Mathematical descriptions of the phenomena in which nanostructures are involved are of great demand because they may be utilized for the purpose of controlling these phenomena (e.g., the growth of nanostructures with certain sizes, shapes, and other properties). Such models may be of distinct nature, including calculations from first principles, ordinary and partial differential equations, and machine learning models (including artificial intelligence) as well. The aim of this article is to review the most important and useful computational and mathematical approaches for the description and control of processes involving nanostructures.

## 1. Introduction

The last few decades have turned the interest of scientists to the phenomena at the nanoscale. Nanoscience has emerged, even if initially it was treated with concerns by common people, and nowadays, it is a very important discipline. One of the topics belonging to nanoscience are the studies of nanomaterials, which deal with materials of sizes lying in the range of 10^−9^ to 10^−7^ m (i.e., from 1 to 100 nm). More useful classification of nanomaterials is based on the exhibition of phenomena that cannot be directly linked with the bulk phase or cannot be described on the molecular level. Such phenomena, which have something in common with both those exhibited by molecules and bulk phases (however, still distinct from them), are characteristics of nanomaterials [1]. This fact contributes to the unique, and often unexpected properties of nanostructures. Such properties determine the wide and interesting applications of nanomaterials. They include, among others, quantum information processing (quantum dots may be used for the preparation of single-photon sources, which may be utilized in quantum computing [2,3]), catalysis (due to the relatively large surface-to-volume ratio of nanomaterials, as well as their high surface energy, e.g., sono- and photocatalysis [4,5]), and biomedicine (fluorescent quantum dots for bioimaging or DNA and RNA nanospheres [6,7,8,9]).

The properties of nanomaterials are crucial for their applications. The prediction of such properties is, thus, a very important issue. Thanks to the development of quantum mechanics, nowadays, it is possible to calculate various properties of nanostructures using density functional theory (DFT) [10,11]. DFT was created by Hohenberg and Kohn in 1964 as a solution for the problem of many interacting bodies; however, a set of assumptions is necessary to use it (despite the exact formalism of DFT) [10,12]. First of all, these assumptions concern the so-called exchange-correlation term: one can choose the local density approximation (LDA, which includes only local quantities of electron density) or the generalized gradient approximation (GGA, which includes the gradient of electron density also) [10]. Secondly, one has to specify the way of treating valence and core electrons [10].

The further development of ab initio methods caused the widespread use of computations in scientific disciplines like chemistry and materials science, including studies of nanomaterials. In consequence, large databases of the calculated properties of various chemical systems were created [10,13]. Thanks to this, it is possible to utilize machine learning algorithms, which are trained on the gathered data [10,13]. As an example, Kuzmanovski et al. presented the capability of artificial neural networks to predict the cell parameters of materials exhibiting symmetry characteristics for perovskite’s structure using the following five input variables: the charge and electronegativity of the anion and the ionic radii of the constituent elements [14]. Then, using the algorithms for the so-called explanation of the machine learning model, the authors determined the effects of distinct input variables on the cell parameters (the target variable) [14].

The recently observed generation of vast amounts of data (both computed and experimental) on materials’ properties (including nanomaterials) led to the creation of new discipline: the so-called materials informatics [10]. An example of action inside this discipline is the Materials Genome Initiative (MGI) started in 2011 and supported by the government of the United States of America [15]. The goal of this initiative is to improve the costs and speed of the discovery, manufacturing, and design of advanced materials [15].

It is obvious that the computational studies of materials, including nanomaterials, are of growing interest. The aim of the presented review article is to present the most recent literature on the application of ab initio and machine learning computational methods for the prediction of the properties of quantum dots and nanostructures.

## 2. First-Principles Studies of Properties of Quantum Dots

In the recent decade, the optical and electronic properties of quantum dots (QDs) have been extensively investigated. The growing popularity of quantum dots can be explained by the possibility of tuning the key parameters, such as the energy states, electron–hole recombination time, photoluminescence quantum yield, absorption coefficient, and adsorption properties. Various first-principle calculations may be utilized to analyze the influence of shape, doping, ligand passivation, electronic defects, and chemical composition, as well as the formation of heterojunctions. Thus, ab initio simulations play a crucial role in designing quantum dot-based materials and processes.

### 2.1. First-Principles Studies of Electronic Properties of Quantum Dots

By applying ab initio calculations, we can gain insights into the electronic properties of quantum dots, such as the energy of states, charge distribution, exciton–phonon coupling, and excited state behavior.

Non-adiabatic TD-DFT calculations are widely used to investigate the properties of excited states. He et al. [16] investigated the influence of the chemical composition of CsPbBr_x_I_y_ QDs on electron–hole recombination via TD-DFT. Nonradiative recombination is the main factor causing a loss of charges, reducing phonon to electron and hole conversion. It was found that an increase in the iodine concentration leads to a longer electron–hole recombination time. The non-adiabatic electron–phonon coupling is weak, and electron–hole coherence is short; this phenomenon is explained by the fact that electrons are supported mainly by Pb atoms, while holes are supported by halogens. In the case of the replacement of bromine atoms with iodine, NA coupling decreases because of lower nuclear velocity. The obtained recombination timescales are in agreement with the experimental data [17].

Long and Prezhdo [18] investigated the electron structure of titania sensitized with methylammonium lead halide perovskite (MAPbI_3_/TiO_2_) with and without doping Cl, Br, and Sn. The calculations revealed that the addition of Cl and Br atoms leads to higher electron delocalization and, therefore, decreases the recombination rate. In contrast, Sn doping raises electron–phonon coupling, leading to an increased recombination rate. This phenomenon is explained by the fact that lighter halogen atoms reduce MAPbI_3_/TiO_2_ interactions because Cl and Br atoms are more strongly connected to Pb, and they cannot form Ti-Hal bonds, while an iodine atom is capable of forming such bonds, providing delocalization, which is unfavorable in terms of electron–hole recombination. In the case of the addition of Sn atoms, Sn-I bonds are shorter than Pb-I, which decreases Ti-I bond formation.

Auger recombination is one of the electron relaxation mechanisms, appearing in systems with a high density of charge carriers, for instance, in semiconducting quantum dots under UV radiation. Trivedi et al. [19] investigated the influence of deep hole traps on Auger-mediated relaxation in order to find a way to minimalize electron relaxation in CdSe quantum dots. Simulations revealed that deep hole traps decrease Auger recombination but do not eliminate it completely. A combination of deep and shallow hole traps can be reached by using different ligands. Such a solution enhances trapping but, at the same time, opens new Auger channels. Therefore, more complex solutions should be examined to design high-performance materials for photocatalytic applications.

**Figure 1:**
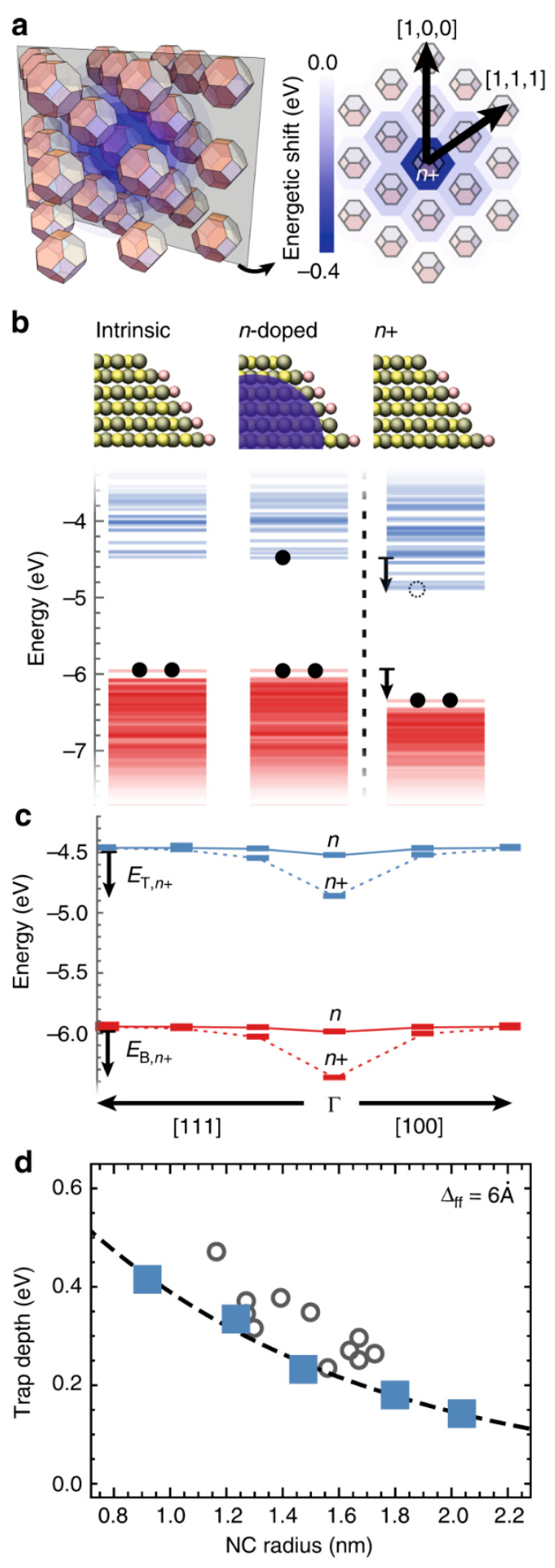
(**a**) A schematic representation of an n+ or p− NC in a solid of intrinsic NCs, where the shift in the energy structure will be screened by neighboring NCs, and a contour plot of the energy shifts of the lowest unoccupied electronic level in NCs (r = 0.95 nm with Δff = 0.6 Å) for an n+ NC in an NC solid. (**b**) Electronic structure of intrinsic, n-doped, and oxidized n-doped NC (n+). (**c**) Highest occupied electronic levels (red) and lowest unoccupied electronic levels (blue) of an n, n+ NC at the Γ point in the BCC lattice and its intrinsic neighbors in the [111] and [100] directions in an NC solid. (**d**) Trap depth as a function of the NC size for NCs in vacuum (circles) and for NC solids (squares). Experimentally measured trap depths on PbS NC solids (grey circles) and the NC charging energies calculated for a sphere of radius r in a PbS NC solid (dashed grey line). Reproduced under the Creative Common license from ref. [20].

Yazdani et al. [20] analyzed the factors influencing charge transport in quantum dots using the example of PbS quantum dots. In comparison to bulk semiconductors, the reorganization energy and electronic coupling of QDs strongly depends on the size of the nanocrystal (NC). It was found that the oxidation of n-doped NCs leads to the formation of electronic traps and barriers for hole transport, while the reduction of p-doped NCs causes the formation of hole traps and barriers for electron transport. A scheme for oxidized n-doped nanocrystals is provided in Figure 1, where n+ denotes oxidized n-doped quantum dots and ∆ff is the facet-to-facet distance. Additionally, the function of the trap depth depending on the NC size is provided. In order to eliminate the traps, the authors suggest the addition of other nanocrystals with a higher energy gap to intrinsic QDs, which prohibits the decrease in effective mobility at high carrier concentrations.

**Figure 2:**
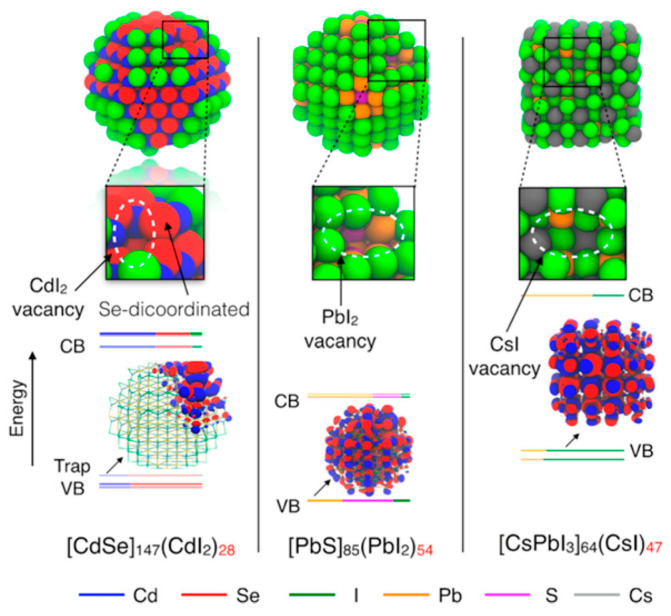
Relaxed structures of three types of QDs (∼2.6 nm) optimized at the DFT/PBE level of theory and with one Z-type ligand displaced: (**left**) a zinc-blende CdSe QD passivated with iodide ions; (**center**) a rock-salt PbS passivated with iodide ions; (**right**) a slightly distorted cubic/orthorhombic CsPbI_3_ perovskite QD. Reproduced under the Creative Common license from ref. [21].

Surface traps play a significant role in nanometric semiconductor properties, being caused by a high ratio of surface atoms to bulk atoms. Giansante and Infante [21] calculated the influence of surface traps on semiconductor properties to analyze the influence on photoluminescence quantum yield (PLQY). Three type of quantum dots were analyzed: CdSe, PbS, and CsPbI_3_. The simulations showed that the elimination of traps is essential for achieving high values of PLQY. Figure 2 presents the energy levels and structures of QDs with the displacement of a Z-type ligand, such as metal complex. CdSe QDs are the most vulnerable to under-coordinated atoms on the surface, supported by the displacement of Z-type ligands. PbS is less affected by this type of defect due to high coordination in the rock-salt type structure, but, at the same time, this type of semiconductor tends to n-type doping. Metal halide perovskites are also resistant to traps caused by Z-type displacement but prone to oxidation, although this behavior may be controlled by applying reducing agents.

Abdelsalam et al. [22] studied the influence of different substituents on the bandgap value of edge-functionalized triangular and hexagonal graphene quantum dots with DFT calculations. Amide, cyano, isopropyl, nitro, and aldehyde groups were placed in zigzag and armchair positions. The analysis demonstrated a larger band gap in the case of armchair–hexagonal, armchair–triangular, and zigzag–hexagonal functionalized GQDs, while zigzag–triangular quantum dots had a smaller energy gap. In all cases, passivation with oxygen significantly decreased the energy gap. The authors studied the total dipole moment of functionalized GQDs, and the largest value was for hexagonal dots functionalized with four aldehyde groups in the armchair position.

**Figure 3:**
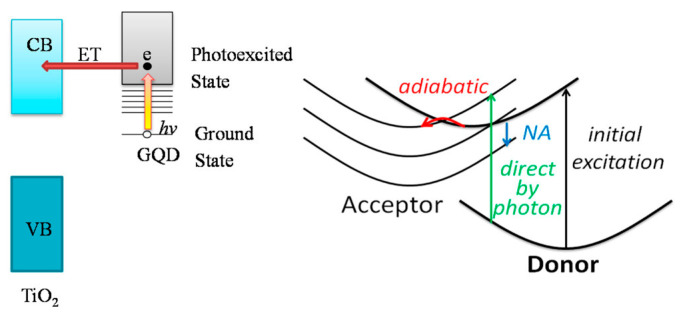
Scheme of photoinduced electron injection from a GQD to the TiO_2_ surface (**left side**). An absorbed photon promotes an electron from the GQD ground state, with energy inside the TiO_2_ band gap, into an excited state that is in resonance with the TiO_2_ conduction band (CB). The excited electron is injected into the TiO_2_ CB via several mechanisms (**right side**). The GQD donor can transfer the electron to the TiO_2_ acceptor either adiabatically by passing over a transition state barrier (curved red arrow) or nonadiabatically via a hop between donor and acceptor states (vertical blue arrow). Additionally, the electron can be promoted from donor to acceptor during the photoexcitation process. Reproduced with permission from ref. [23].

Long et al. [23] investigated GQD/TiO_2_ composites to analyze photoinduced electron behavior and, in particular, donor–acceptor coupling and electron transfer (ET). Schematic illustrations are provided in Figure 3. Stronger donor–acceptor coupling enhances the rate of adiabatic ET. In contrast, relatively weak donor–acceptor coupling causes a higher non-adiabatic ET rate. It was revealed that donor–acceptor coupling in the studied systems is mainly caused by π-electron interactions of TiO_2_ with GQDs, while covalent bonds play a minor role in this phenomenon. Additionally, it was found that the influence of a so-called linker group, which responds to the bonding of GQDs with TiO_2_, is relatively slow in comparison to the π-electron system. Moreover, bigger π-electron systems cause stronger donor–acceptor coupling. The calculations showed that vibrations during ET are mainly caused by GQDs, which have lighter atoms than TiO_2_. Analyses of photo-excited states led to the conclusion that in the described system, charge separation occurs faster than energy relaxation, which is fundamental for the use of GQDs/TiO_2_ in photocatalysis and photovoltaics.

### 2.2. First-Principles Studies of Optical Properties of Quantum Dots

The optical properties of nanocrystals, such as light absorption, emission, Stokes shift, and others, can be simulated using quantum mechanical calculations. This subsection will discuss case studies on this topic.

The calculated properties of chalcogenide quantum dots can be different from the experimental results because of the chemical impurities causing the appearance of defect-induced in-gap states (IGSs). Zhang et al. [24] investigated, theoretically, what the source of such defect-induced in-gap states (IGSs) in PbS quantum dots is. Density of states analysis was performed using DFT calculations in order to analyze different potential defects. During the DFT calculations, the authors utilized VASP software and generalized gradient approximation with a PW91 functional and also a hybrid HSE03 functional. The results showed that the adsorption of PbCl_2_, O^2−^, H_2_O, and dissociated oxygen does not produce IGSs. The main factor responding for IGSs is molecular oxygen, O_2_. Taking into consideration this fact, it is possible to create high-efficiency optoelectronic devices.

Liu et al. [25] researched Franck–Condon (FC) shifts of PbS quantum dots with both experimental methods and DFT simulations. It was inferred from both calculations and photoluminescence experiments that higher electronegativity of ligands leads to higher FC shifts; however, the effect is small. Additionally, a Stokes shift in polydisperse PbS solid film is higher than in the case of isolated quantum dots. The main effect causing excessive FC shifts and, consequently, a Stokes shift are intrinsic electronic defects, and the highest value was found in the case of V^+^_Cl_.

The influence of heteroatom doping on graphene quantum dots (GQDs), the HOMO-LUMO gap, and electron density was investigated using TD-DFT by Feng et al. [26]. The calculated adsorption of undoped GQDs is consistent with experimental data. Different positions of heteroatoms were considered (basal plane, hexatomic ring, and pentatomic ring at edge). In all cases, doping caused a decrease in absorption, nitrogen and sulfur doping caused a red-shift, and boron and phosphorus exhibited a blue-shift. A change in the HOMO-LUMO energies is strongly connected to doping with heteroatoms, making bandgap tuning possible. Charge difference density analysis revealed that heteroatoms support charge transfer, which, in the excited state, increases the HOMO-LUMO gap. It was discovered that atoms with a relatively large radius doped on a basal plane transform sp^2^ hybridized carbon atoms into sp^3^, changing the geometry of the particle. Later, deeper analyses were conducted by the same group [27] in order to investigate the optical properties of surface- and edge-doped GQDs with different boron species. In all the studied systems, each boron atom’s coordination number is three. Doping with unoxidized boron when a boron atom is bonded with three carbon atoms is named BC_3_, and oxidized boron connected to carbon atoms of graphene and one or two -OH groups is noted as BC_2_O and BCO_2_, respectively. Unoxidized boron doping causes a significant decrease in absorption, which is consistent with previous reports [26]. In general, oxidized boron species exhibit lower influence on GQDs and lead to a lower decrease in absorption and even increase in the case of edge-doped BCO_2_. It is interesting that edge-doped BCO_2_ demonstrated a mild red-shift, while surface-doped BCO_2_ demonstrated a blue-shift. Excited state analyses displayed that BC_2_O-doped GQDs show delocalization with a similar electronic transition behavior as undoped ones, while BCO_2_ exhibits localization on the graphene plane. Feng et al. [28] also investigated the influence of five oxygen-containing groups on the optical properties of graphene quantum dots. It was revealed that edge functionalization almost does not influence the optical properties of materials, while surface modification causes a decrease in absorption and red-shift. The highest red-shift is observed in -COOH surface-functionalized quantum dots. It is interesting that surface modification with an epoxy group caused the highest absorption in comparison to other groups, while the maximum wavelength almost did not change. The changes are strongly connected with the electronic structure. An analysis of the molecular orbitals and charge transfer revealed that electronic transition is locally excited, and oxygen-containing groups leads to lower electron localization.

### 2.3. First-Principles Studies of Adsorption Properties of Quantum Dots

DFT calculations are widely used to investigate intermolecular interactions. The high specific surface area and electronic structure of quantum dots make this type of material a potentially good adsorbent.

**Figure 4:**
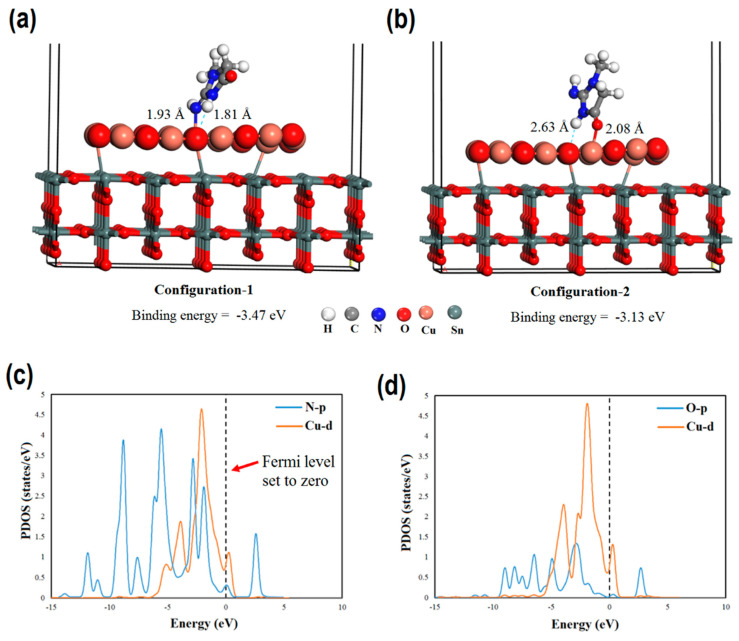
(**a**) Optimized geometry of SnO_2_@Cu_2_O nanostructure electrode, (**b**) side view, and (**c**) corresponding total density of states plot and (**d**) PDOS plot. Reproduced under the Creative Common license from ref. [29].

Ullah et al. [29] analyzed the Cu_2_O-decorated SnO_2_ nanoparticles in terms of creatinine detection. DFT simulations were applied to study the adsorption properties and selectivity. Two adsorption configurations were analyzed, and the optimized geometries are provided in Figure 4: in the first, N and H atoms from the amine group of the creatinine molecule were oriented toward the Cu and O atoms of the nanostructure, respectively; in the second, the O atom from the carbonyl group and the H atom from the amine group were directed toward the Cu and O atoms in the nanoparticles. It was revealed that the absolute value of binding energy was higher in the first configuration, with a value of 3.47 eV. In order to investigate selectivity, various molecules were analyzed, and the absolute value of binding energy of all the considered molecules decreased in the series: creatinine > NHS > uric acid > glucose > urea > L-cysteine > dopamine > ascorbic acid > cholesterol. The closest value of binding energy was in case of N-Hydroxysuccinimide (NHS), and the difference between binding energies was 0.6 eV, which is a significant difference, suggesting the high selectivity of SnO_2_/Cu_2_O nanostructures in terms of creatinine adsorption.

Montejo-Alvaro et al. [30] studied, theoretically, the interactions between small gas molecules and doped graphene quantum dots. Calculations were performed using BLYP with NL and D3 corrections, and it appears that the NL correction results are closer to experimental data. The doping of graphene increases the binding energy with water, carbon monoxide, and ammonia molecules. Higher binding energy is caused by interactions of lone pair of gas molecule with Al and Si doping. B- and N- doped graphene quantum dots interacted with gas molecules via van der Waals forces.

The adsorption properties of graphene can be used to develop drug delivery systems. Vatanparast and Shariatinia [31] performed drug adsorption calculations using DFT with Grimme’s dispersion corrections in order to use doped and undoped graphene quantum dots (GQDs) in drug delivery systems for a 5-fluorouracil (FU) drug. The pristine GQD was hexa-peri-hexabenzocoronene (HBC, C_42_H_18_). In doped GQDs, the three C-C bonds were replaced with Be-N, Be-P, Al–N, and Al–P bonds, forming a C_36_X_3_Y_3_H_18_ structure (where X is B or Al and Y is N or P), which were named as BN, BP, AlN, and AlP GQDs, respectively. The results of the comparison of adsorption rates for the doped GQDs were as follows: AlN-FU > AlP-FU > BP-FU > BNFU, with the most negative energy value of −47.29 kcal/mol. Overall, it was proven theoretically that doped GQDs could be used as a 5-fluorouracil drug carrier.

**Figure 5:**
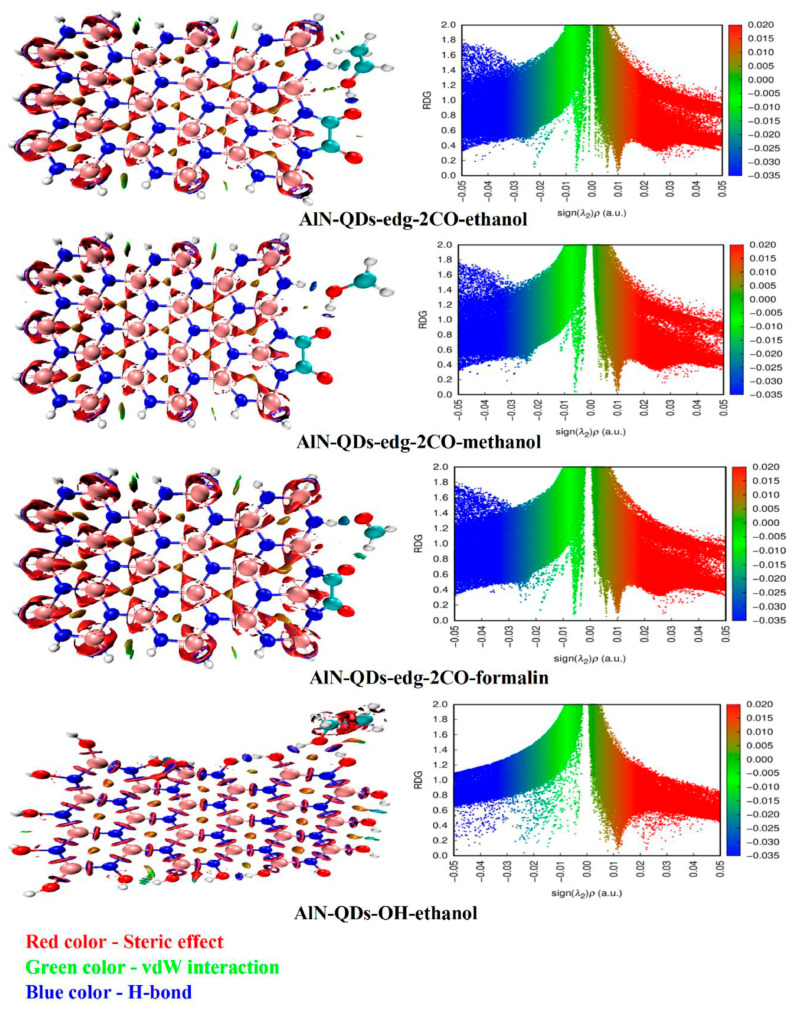
NCI surfaces (**left**) and scatter graph (2D graph) (**right**) of AlN-QDs-edg-2CO-ethanol, AlN-QDs-edg-2CO-methanol, AlN-QDs-edg-2CO-formalin, and AlN-Qds-OH-ethanol. Reproduced under the Creative Common license from ref. [32].

Saad et al. [32] investigated 2D AlN quantum dots and their functionalized derivatives with first-principles calculations. Edge-functionalized aluminum nitride with two CO groups, named AlN-QDs-edg-2CO, and functionalized with OH groups at all edge positions (AlN-QDs-OH) were chosen as the most stable particles for the adsorption study. The adsorption strength grows in the following order: AlN-QDs-edg-2CO-formalin < AlN-QDs-edg-2CO-methanol < AlN-QDs-edg-2CO-ethanol < AlN-QDs-OH-formalin < AlN-QDs-OH-methanol < AlN-QDs-OH-ethanol. Non-covalent interaction (NCI) analysis was performed to investigate the influence of a wide range of intermolecular interactions. The results are provided in Figure 5, where a two-dimensional plot presents the reduced density gradient (RDG) versus (λ2)ρ. Values of ρ > 0 demonstrate attractive interactions, and ρ < 0 shows repulsive interactions. The three-dimensional plot highlights the iso-surface topology. The theoretical study revealed the excellent adsorption properties of the chosen, functionalized 2D AlN QDs, suggesting their potential use in sensors.

### 2.4. First-Principles Studies of Catalytic Properties of Quantum Dots

DFT calculations are an important tool for the analysis of the mechanism of catalytic processes. The Gibbs free energy of sequent steps determines the rate of the whole reaction and can be analyzed using first-principles-based computational methods.

Sheng et al. [33] discovered the catalyst enabling the eight-electron reduction of CO_2_ to CH_4_ and analyzed the mechanism of this reaction. The photocatalytic activities of Cs_2_CuBr_4_ and CsPbBr_3_ quantum dots (QDs) for the reduction of CO₂ to CO and CH₄ were examined. DFT calculations, performed using VASP software, and GGA-PBE approximation (the densities of the states were calculated using the HSE06 hybrid functional) suggest that the replacement of the Pb atoms present in traditional CsPbBr_3_ halide perovskites with Cu led to a d-band closer to the Fermi level, causing better CO_2_ adsorption. The limiting step of this process is the formation of the adsorbed COOH intermediate, and a huge decrease in activation energy was observed in the case of Cs_2_CuBr_4_. In addition, after CO formation, for CsPbBr_3_, the energetically desired route is CO desorption, which prevents further reduction to methane, while in the case of Cs_2_CuBr_4_, methane formation is a result of a step-by-step energy-favored route, leading to the eight-electron reduction of CO to CH_4_, which is proven experimentally [33].

Liu et al. [34] investigated, both experimentally and theoretically, ZnO/reduced graphene oxide (RGO) composites to develop a catalyst for the electrochemical reduction of nitrogen to ammonia. DFT calculations were conducted to obtain more data about the mechanism of reaction. The slowest step, determining the whole process rate, is the hydrogenation of adsorbed nitrogen to *N_2_H. Analyses revealed a significant decrease in Gibbs free energy during these steps in the case of ZnO/RGO in comparison to pristine ZnO. The experiments are consistent with calculations showing an NH_3_ yield of 17.7 μg·h^−1^ ·mg^−1^ with a Faraday efficiency (FE) of 6.4% at −0.65 V. The same group [35,36] conducted similar experiments with CoO/RGO and SnO/RGO. CoO/RGO demonstrated an NH_3_ yield of 21.5 μg·h^−1^ ·mg^−1^ with an FE of 8.3% at −0.6 V. In the case of SnO_2_, the results are 25.6 μg h^−1^ mg^−1^ and an FE of 7.1% at −0.5 V.

Graphitic carbon nitride can be used in catalytic water splitting, but its bandgap value of 3.02 eV is too high for visible light adsorption, so Gao et al. [37] suggested using carbon dots/graphitic carbon nitride (CD/g-C_3_N_4_) heterojunction. DFT calculations (based on the GGA-PBE and HSE06 functionals) were used to investigate the effect of the decoration of g-C_3_N_4_ with carbon dots. It was calculated that the energy of the carbon dots’ HOMO orbital is in the middle between the HOMO and LUMO orbital energies of carbon nitride, so carbon dots form a type-II Van der Waals heterojunction, which can be used to improve the photocatalytic water-splitting process.

Mohanty et al. [38] synthesized MoS_2_ quantum dots for a catalytic oxygen evolution reaction (OER). First-principle calculations (based on DFT with the RPBE exchange-correlation functional implemented in the Atomistix ToolKit) proved that the OER is more favorable on the edge and vertex positions, while the surface positions are almost inert. Edge defects such as sulfur vacancies cause increase with catalytic performance. This reaction is more favorable on the vertex positions than on the edge positions. With no external potential, the OER is not energetically favorable, and the limiting step for such a process on the surface and edge positions is the reaction between OH^-^ and the adsorbed oxygen atom, while on the vertex position, it is oxygen desorption. Additionally, the free energies of the steps were calculated for the electrochemical process with an external potential of 0.6 V and pH = 14, which is required to make the whole oxygen evolution reaction an exothermic process. In such a process, the limiting step for edge positions is oxygen desorption. In brief, sulfur vacancies enhance the catalytic activity, the whole process appears mostly on the edge and vertex positions, and the limiting step for the process with external potential of 0.6 V is O_2_ desorption.

DFT calculations were used by Wang et al. [39] to understand the origin of enhancement of the photocatalytic performance of BiSbO_4_ QDs on a carbon dot (CD) composite in comparison to pristine BiSbO_4_ QDs. The authors used GGA-PBE approximation during the DFT simulations. It was revealed that carbon atoms form an electron depletion layer on the inner side of BiSbO_4_ with p-type CDs and n-type BiSbO_4_. Additionally, O_2_ molecules adsorbed on carbon dots are prone to electrons on the CD-BiSbO_4_ layer, creating good conditions for O_2_^−^ anion radical creation. The hypothesis is consistent with the XPS data [39].

A summary of the described chemical systems studied with the DFT calculations is presented in Table 1.

## 3. Machine Learning for Prediction and Utilization of Properties of Quantum Dots

### 3.1. Machine Learning-Assisted Investigations of Carbon Dots

The majority of the most recent literature on applications of machine learning in the investigations of nanostructures concerns carbon dots (CDs). Despite their similar sizes lying in the range of 1–10 nm, generally, CDs may be divided into four distinct groups (however, this categorization is still under discussion): carbon quantum dots (CQDs) which are quasi-spherical in shape and crystalline; graphene quantum dots (GQDs), which have discoidal shapes and are crystalline but less than CQDs; carbon nanodots (CNDs), which are quasi-spherical in shape but amorphous; and carbonized polymer dots (CPDs), which also have a quasi-spherical shape, but their crystallinity is not well defined and may lie somewhere between that of CQDs, GQDs, and CNDs, depending on the situation [40,41,42]. Carbon dots find varied applications, typical for nanomaterials, such as biosensing, drug delivery, cancer therapy, optoelectronics (e.g., LEDs), photocatalysis, and others [40,41,42]. CDs have many advantages: their synthesis is relatively simple and uses easily accessible compounds (such as citric acid, gallic acid, ascorbic acid, and dopamine); they are stable in long-term conditions; and they are biocompatible, biodegradable, and relatively low toxic (or even non-toxic) [40,41,42]. The synthesis of carbon dots, as already mentioned, is relatively straightforward; however, the control of the product is difficult due to the possibility of passivation of the CD surface by various organic functional groups (amino-, carbonyl-, aldehyde-, epoxy-, hydroxyl-, carboxylic groups, etc.) and by the incorporation of sp^2^ and sp^3^ carbon atoms in the carbon core of CDs in variable proportions [40,41,42]. These problems significantly contribute to the wide usage of machine learning approaches in the studies of the synthesis and applications of carbon dots.

He et al. studied the synthesis of carbon dots for the purpose of corrosion inhibition [43]. Due to the relatively good solubility of CDs in water, as well as due to their relatively low impact on the environment and high chemical inertness, CDs are candidates for next-generation green corrosion inhibitors; however, the controllable synthesis of CDs with the desired inhibition efficiency is hard to achieve [43]. He et al. applied machine learning algorithms to correlate the hydrothermal synthesis parameters with the inhibition efficiency. Their dataset consisted of 102 experimental points in total, collected from the literature and own studies [43]. The input variables were the CD content, precursor type (citric acid, ammonium citrate, or o-phenylenediamine), solvent type and its volume, and reaction time and temperature. Among machine learning models, the authors compared Gaussian process regression (GPR), K-nearest neighbors (KNN), XGBoost, support vector regression (SVR), and random forests (RFs). The latter was found to be the best in terms of prediction accuracy, which was greater than 90% [43]. Then, using the trained RF model, a genetic algorithm (GA) was used to successfully perform the optimization of the synthesis process: the measured inhibition efficiency of the CDs prepared under the optimal conditions proposed using a GA was 92.3% versus theoretical 94.6% [43].

Due to the fluorescence properties of carbon dots, they are widely investigated also in the field of sensitive detection. For example, Pandit and cooperators utilized surface-functionalized CDs for the sensing of proteins [44]. The greatest problem in such sensing is the analysis of the optical pattern generated by the sensor array. Pandit et al. overcame this issue by utilizing the pattern recognition ability of machine learning algorithms [44]. The authors tried seven distinct types of machine learning algorithms: multinomial logistic regression (MLR), Naive Bayes (NB), support vector machines (SVMs), K-nearest neighbors, decision trees (DTs), gradient-boosted trees (GBTs), and random forest [44]. The investigated proteins were lysozymes, cytochrome c, alpha-chymotripsin, hemoglobin, lipase, alpha-amylase, bovine serum albumin, and beta-galactosidase; each of these proteins had a significantly distinct fluorescence pattern [44]. Four of the seven studied machine learning techniques achieved 100% differentiation between the proteins of interest, outperforming the statistical method, which was linear-discriminant analysis [44].

Another study on the sensing application of carbon dots (precisely carbon quantum dots in the following case) is the detection of glucose. Ullah khan and cooperators utilized CuO modified with CQDs for that purpose [45]. Metal oxide structures are prone to surface passivation and are characterized by poor conductivity; thus, the authors are seeking to improve the electrocatalytic properties of CuO by depositing CQDs on its surface [45]. Four distinct machine learning models were applied to correlate the glucose detection abilities; they were linear regression (LR), random forest, LightGBM, and an artificial neural network (ANN). The latter outperformed the rest of the machine learning algorithms [45].

Das et al. utilized Au–nitrogen-doped GQD-based heterostructures (Au@N-GQDs) for metal ion sensing [46]. The authors used the data produced by the constructed photodetector, containing the aforementioned nanoparticles, to train and test a variety of machine learning algorithms (the above 20 algorithms, including support vector machines, neural networks, Gaussian process regression, linear regression, and decision trees), and the best performing algorithm was chosen for the optimization of the working wavelength of the photodetector [46]. A two-step machine learning approach was used. In the first step, the authors correlated the spectral responsivity of the photodetector with the wavelength of incident light, input power, and photocurrent. In the second step, the predicted (by machine learning algorithm trained in the first step) values of spectral responsivity and the wavelength of incident light were used as input variables for the prediction of the external quantum efficiency [46]. Thanks to such a two-step approach, a trained machine learning model is obtained that is able to optimize the conditions of the photodetector [46]. Then, in such conditions, the selectivity of the photodetector toward various metal ions was investigated. In this case, the inputs for the machine learning model were the light current, dark current, and intensity ratio of the photocurrents (before and after the addition of metal ions; I_0_/I), while the output was the name of the metal ion [46]. Having the classification algorithm trained, it was further used to determine the metal ion with the greatest selectivity, which was Fe^3+^ cation [46]. For this ion, predictions of sensitivity using machine learning were conducted, taking the following physical quantities as input variables: light current, dark current, and the concentration of the metal ion; the ratio of photocurrent (I/I_0_) was the output variable [46]. The combined approach of using Au@N-GQDs with a machine learning algorithm proved its applicability in the detection of the Fe^3+^ cation in real-world samples from Brahmaputra River [46].

**Figure 6:**
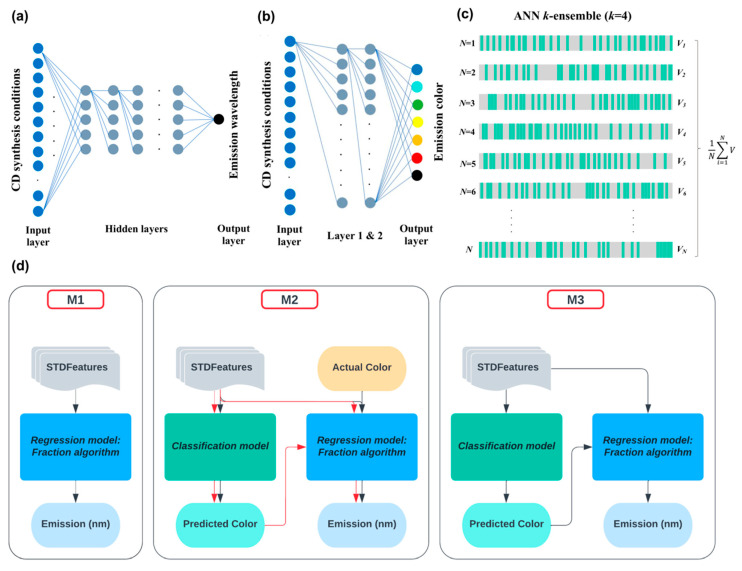
Summary of machine learning models, their algorithm, and training workflows in the study of Senanayake et al. [33]. (**a**) Artificial neural network (ANN) structure with input, hidden, and output layers used to predict the emission wavelength through the regression model. (**b**) ANN structure with input, two hidden, and output layers used to predict the emission color in the classification model, with only a few connections shown between nodes in the ANN for simplicity. (**c**) ANN k-ensemble (k = 4). (**d**) Training workflow of the three ML models (black arrows). M1 is the regression model, M2 is the hybrid model with classification and regression in parallel, and M3 is the hybrid model where regression model training follows classification training. The testing workflows in M1 and M3 are similar to their training workflows. In M2, the testing workflow is different from the training workflow and testing workflow, as indicated by the red arrows. Reproduced under the Creative Commons license from ref. [48].

As seen from the examples described above, the fluorescence properties of carbon dots are crucial for their applications. Machine learning was widely used for the purpose of the prediction of fluorescence properties. Xing et al. applied six distinct machine learning algorithms (random forest, k-nearest neighbors, decision trees, support vector machines, AdaBoost, and Bagging) to predict the photoluminescence peaks of the strongest intensity and the longest wavelength on the basis of precursor combinations at two different excitation wavelengths (365 and 532 nm) [47]. The random forest model outperformed other machine learning algorithms, and it is speculated that it may be used in the effective screening of CDs with desired photoluminescence properties, in contrast to time-consuming experimental random trials [47]. Another study presented by Senanayake et al. utilized only one machine learning algorithm, specifically an artificial neural network, with the purpose of correlating the parameters of synthesis of carbon dots with the emission color and wavelength of CDs [48]. The performed modeling showed that the choice of reaction method, purification method, and solvent has greater influence on the CD emission characteristics than the parameters commonly varied in experiments, such as time and temperature [48]. The authors also studied k-ensembles of artificial neural networks, as well as a two-stage approach, similar to that described in one of above-mentioned studies (see Figure 6) [48].

The authors used three different machine learning architectures, denoted as M1, M2, and M3 [48]. M1 stands for a single artificial neural network applied for regression, while M2 and M3 are hybrid machines, consisting of two ANNs in series. These models consisted of a classification model for color prediction and a regression model for wavelength prediction, while the color predicted by the classifier was used as the input variable for the regressor [48]. The hybrid machines achieved a higher accuracy than the single ANN (M1). It was also found that incorporating the time and temperature of synthesis into the input features improves the overall prediction. In the hybrid machines, the performance was also improved by using an ensemble of classifiers instead of one classifier [48].

**Figure 7:**
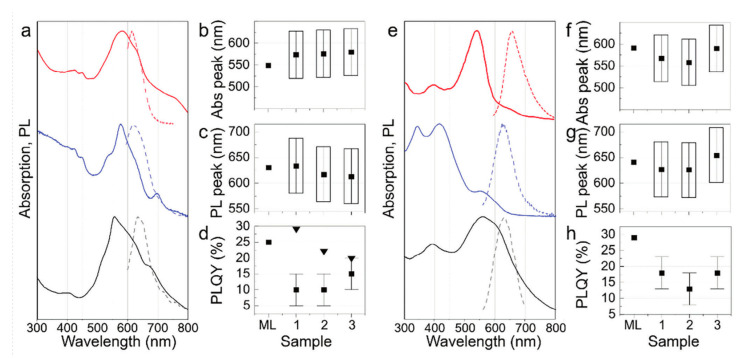
Comparison of predicted and experimental properties of CD-1 (**a**–**d**) and CD-2 (**e**–**h**) samples obtained in three different laboratories: 1—black, 2—blue, 3—red; (**a**,**e**) absorption (solid lines) and photoluminescence (dashed lines) spectra; (**b**,**f**) absorption and (**c**,**g**) photoluminescence peak positions, and (**d**,**h**) photoluminescence quantum yields. Boxes indicate full widths at half maximum. Reproduced with permission from ref. [49].

Tuchin et al. created and analyzed a dataset on the synthesis parameters and optical properties of carbon dots, with a focus on optical transitions in the red and near-infrared spectral ranges [49]. The authors used multiple linear regression as the machine learning model, and the results were verified by a comparison of the predicted data and data experimentally obtained in three different laboratories [49]. The dataset was constructed following an analysis of 110 research articles and 127 syntheses of CDs; it included information on the synthesis method (solvothermal or microwave assisted), type of precursor, type of solvent, mass/volume ratio of precursor to solvent, time, temperature, power (for syntheses using microwaves), positions of the absorption and emission maxima in longer-wavelength spectral regions, and values of photoluminescence quantum yields (PLQYs) [49]. The optical parameters were identified as the target variables for machine learning. The authors used a multiple linear regression (LR) function of the form:

(1) $$Target=a\cdot M_{1}+b\cdot M_{2}+c\cdot M_{3}+d\cdot T+e\cdot t+f$$

in which *Target* is the absorption or PL peak position, or PLQY, *M_i_* is the molar concentration of the i-th precursor (in mmol/mL), *T* is the temperature (in °C), *t* is the time of synthesis (in h), and *a*, *b*, *c*, *d*, *e*, and *f* are numerical coefficients [49]. The authors justify the use of linear regression based on the simplicity of this model and easy avoidance of overfitting; other machine learning models were also tested (random forest, decision trees, and k-nearest neighbors); however, they were prone to overfitting on small datasets [49]. As already mentioned, the applicability of the trained machine learning model used was experimentally checked via a comparison with data obtained in three different laboratories (1—ITMO University in Russia, 2—City University of Hong Kong, and 3—Jilin University in China) on the examples of two syntheses of red-emissive CDs: the first (denoted as CD-1) starting from citric acid (CA) performed in formamide (FA) and the second (denoted as CD-2) starting from citric acid and also from urea in dimethylformamide (DMF). Figure 7 shows a comparison of the experimental and predicted data. The validation proves the relatively good agreement between the predicted and experimental data [49].

Another application of machine learning methods was presented by Dager and cooperators, who utilized ML for the analysis of photoluminescence of mono-disperse carbon quantum dots from fennel seeds [50]. CQDs prepared via single-step thermal decomposition of fennel seeds are characterized by relatively good colloidal, photo-, and environmental stabilities [50]. Moreover, their surface does not need to be passivated to improve their fluorescence. Such CQDs had relatively good photoluminescence activity and excitation-independent emission [50]. The authors used the machine learning techniques PCA (principal component analysis), MCR-ALS, and NMF-ARD-SO to elucidate the mechanism of photoluminescence of the studied CQDs. The machine learning algorithms allowed for the selection of the best excitation wavelength for photoluminescence analysis [50].

**Figure 8:**
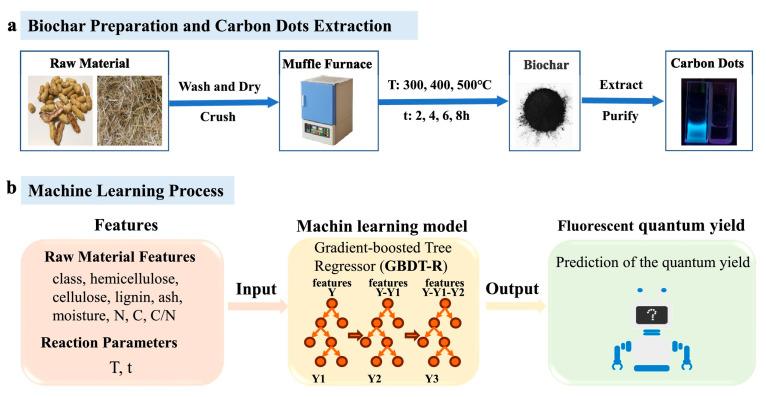
A general scheme of machine learning-assisted CQD extraction from biochar [36]. (**a**) Extraction of CQDs from biochar to establish a training dataset for machine learning; (**b**) machine learning prediction of the quantum yields of CQDs extracted from biochar. Reproduced with permission from ref. [51].

Chen et al. utilized machine learning algorithms to predict the photoluminescence quantum yield of carbon quantum dots separated from biochar originating from 10 types of farm waste [51]. The authors used ML for the generation and extraction of CQDs from biochar in two steps. In the first step, the authors identified 11 parameters for the biochar-production experiment: the type of farm waste, cellulose content, hemicellulose content, lignin content, ash content, moisture content, N content, C content, C/N ratio in the samples, pyrolysis temperature T (chosen from 300, 400, and 500 °C), and residence time t (chosen from 2, 4, 6, and 8 h) [51]. In total, the dataset contained information on 480 CQD samples. The target variable was the value of photoluminescence quantum yield, and the dataset was randomly divided into training and validation datasets with a ratio of 8:2. The validation dataset was used for the preliminary check of the effectiveness of the trained algorithm. Figure 8 presents the general scheme of the procedures adopted for the experiments and calculations [51].

**Figure 9:**
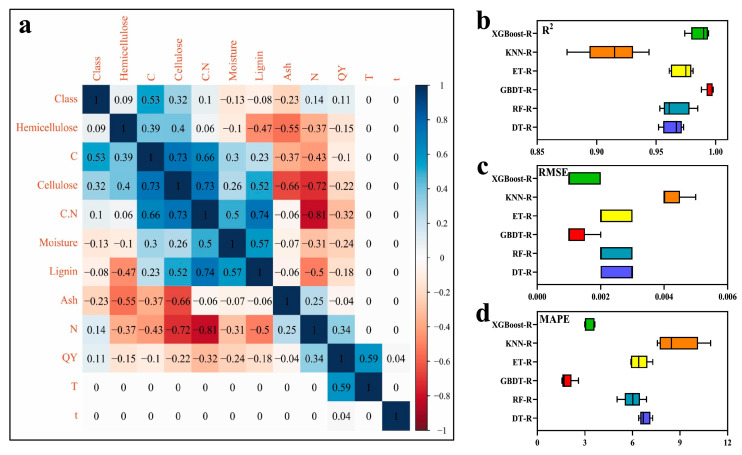
Scheme of construction of ML model in ref. [51]. (**a**) The heat map of Pearson’s correlation coefficient matrix among the selected features of CQDs produced during biochar preparation; (**b**) comparison of coefficient of determination values (R^2^) for studied ML models; (**c**) RMSE values for studied ML models; (**d**) MAPE values for studied ML models. Reproduced with permission from ref. [51].

The authors used Pearson’s correlation statistical analysis to check for linear correlations in pairs of the selected 11 variables. The type of machine learning was regression, and the following regression models were used: decision tree regression (DT-R), random forest regression (RF-R), gradient-boosting decision tree regression (GBDT-R), extra trees regression (ET-R), K-nearest neighbors regression (KNN-R), and XGBoost regression (XGBoost-R) [51]. The GBDT-R model was chosen as the algorithm, based on which the feature importances were determined. The selection of this model was justified by values of the R^2^, RMSE (root-mean-square error), and MAPE (mean absolute percentage error), which were the best among the six investigated models (R^2^ > 0.9, RMSE < 0.02, and MAPE < 3; see Figure 9). On the other hand, the Pearson correlation analysis justified the selection of input and output features (Figure 9) [51].

The trained GBDT-R model was used for the extraction of feature importances based on the training and validation data [51]. It was found that there were 4 (from among 11) most important features: the pyrolysis temperature, residence time, N content, and C/N ratio [51].

**Figure 10:**
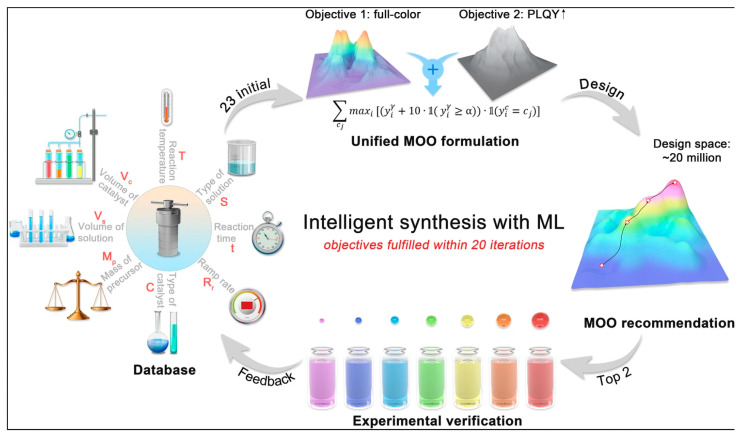
Scheme of workflow of ML-guided investigation of synthesis of CQDs performed in ref. [52]. Reproduced under the Creative Common license from ref. [52].

Guo and cooperators incorporated machine learning methods in the studies of hydrothermal/solvothermal synthesis of CQDs [52]. The optimization of the product of such a synthesis is challenging using random experimental trials due to the numerous synthesis parameters [52]. The authors used ML-integrated MOO (multi-objective optimization) strategy to learn the complex relations between the synthesis parameters and two target properties (full-color photoluminescence wavelength and photoluminescence quantum yield) of CQDs [52]. Thanks to this, it was possible to determine the optimal conditions for both properties simultaneously. Figure 10 shows the overall scheme of the workflow of ML modeling in the study [52]. This workflow consists of four main components: the construction of the database, the formulation of multi-objective optimization, MOO recommendation, and, lastly, the experimental verification [52].

Additionally, the authors used the trained machine learning model to synthesize full-color fluorescent CQDs with tunable photoluminescence spanning from blue to red light, using only 63 experiments guided by the ML model [52].

**Figure 11:**
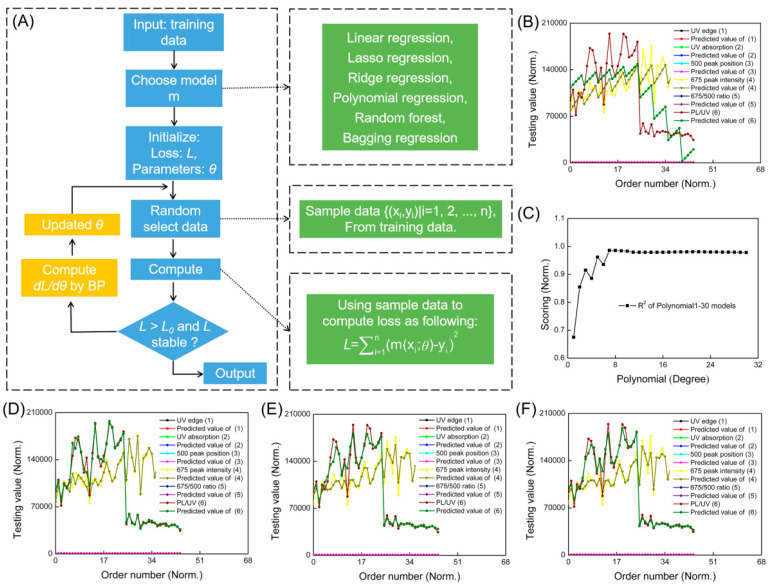
Scheme of performed machine learning modeling of B,N-GQD properties [53]. (**A**) Schematic of machine learning-assisted evaluation of optical properties of B,N-GQDs. Optical properties of B,N-GQDs in varied synthesis conditions and corresponding predicted value sets with (**B**) linear regression; (**C**) polynomials 1–30; (**D**) polynomial regression 7; (**E**) bagging regression; and (**F**) random forest regression. Reproduced with permission from ref. [53].

Zhang et al. studied the synthesis of boron- and nitrogen-doped GQDs (B,N-GQDs), performed via the oxidation of 3-aminophenylboronic acid monohydrate in the hydrothermal reaction vessel [53]. Machine learning was used to investigate the influence of synthesis conditions—synthesis temperature, additional H_2_O_2_ volume, and synthesis time—on the resulting products [53]. The products were characterized using many techniques, among which UV–Vis and PL spectroscopies were used to derive the optical parameters, which were then used as target variables in machine learning [53]. These optical parameters included UV spectrum edge position, UV absorption intensity, photoluminescence peak position in the range of 400–600 nm, peak intensity at 675 nm, 675/500 peak intensity ratio, and PL/UV intensity ratio [53]. Among the machine learning algorithms used by the authors, the following may be listed: linear regression, polynomial regression, lasso regression, random forest regression, bagging regression, ridge regression, and more [53]. The weights of the best ML models were analyzed to elucidate the influence of synthesis conditions on the optical properties of the product, and the same model was utilized in the optimization process performed with the greedy random walk algorithm [53]. Figure 11 presents the scheme of the applied procedure in the machine learning study. The value of the R^2^ coefficient for the linear regression model was 0.6751, which is much lower than the industrial standard value (0.8). On the other hand, the R^2^ coefficient values for polynomials of orders 2 and 7 were 0.8548 and 0.9860, respectively [53]. The authors performed a careful selection of the evaluation parameter, for which the 675/500 intensity ratio was finally chosen. For the determination of the influence of synthesis conditions on the mentioned evaluation parameter, the polynomial of degree 7 was chosen [53]. It turned out that the additional H_2_O_2_ volume was the most important parameter in the synthesis of B,N-GQDs, indicated by the value of weights for each synthesis parameter [53]. The authors explain that the positive influence of H_2_O_2_ addition is due to its decomposition into O_2_, H_2_O, a hydroxyl radical, and a hydroperoxyl radical, from which the high level of reactive free radicals accelerate the crystallization and dispersibility of B,N-GQDs in aqueous solution [53]. The authors also used the best machine learning model to optimize the synthesis conditions based on the greedy random walk algorithm, and the sample prepared at the optimal conditions exhibited a relatively high 675/500 peak intensity ratio (0.285) and photoluminescence quantum yield (0.74%) [53].

**Figure 12:**
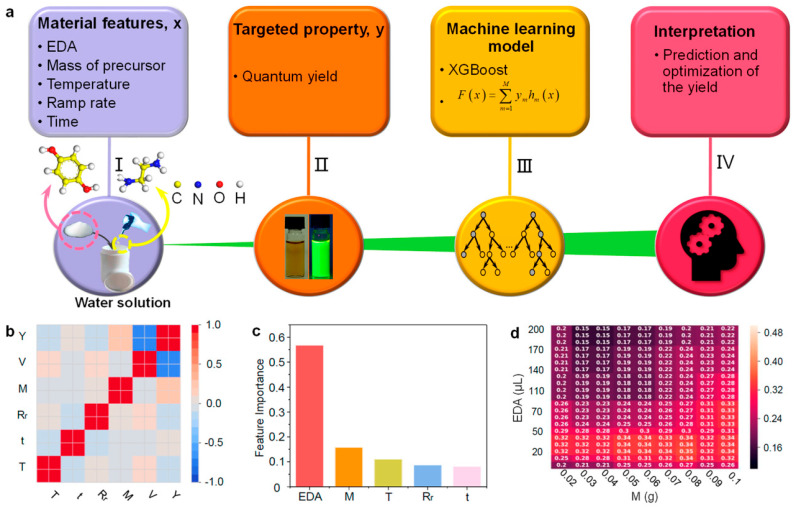
Scheme of performed machine learning modeling of hydrothermally derived CD properties [54]. (**a**) Design framework for the guided synthesis of CDs with a large QY based on ML and hydrothermal experiments. (**b**) The heat map of Pearson’s correlation coefficient matrix among the selected features of hydrothermal-grown CDs. (**c**) Feature importance retrieved from XGBoost-R that learns from the full dataset. (**d**) Predictions from the trained model, which is represented by the matrix formed by the two most important features. Reproduced with permission from ref. [54].

Another study of the influence of synthesis conditions in hydrothermal synthesis on the properties (especially on the fluorescent quantum yield) of synthesized carbon dots was performed by Han et al. [54]. Thanks to the applied machine learning, the authors were able to obtain CDs with a strong green emission characterized by a quantum yield up to 39.3% [54]. Also, the authors were able to determine the influence of distinct synthesis parameters, finding that the mass of the precursors and the volume of alkaline catalysts were the most important features [54]. Figure 12 shows the scheme of the performed machine learning modeling. Prior to ML modeling, the authors identified the five most significant experimental parameters that were used as input features for the ML model: the volume of ethylenediamine (EDA), mass of the precursor, reaction temperature, ramp rate, and reaction time [54]. In total, 391 experiments were conducted, and for each sample, the quantum yield (output variable in the ML modeling) was measured. As shown in Figure 12b, the selected features were not linearly correlated (in pairs). A nested cross-validation was applied, and the following machine learning models were used: XGBoost regressor, multilayer perceptron regressor (MLP-R), support vector machine regressor (SVM-R), and Gaussian process regressor (GP-R). The ML models were evaluated basing on the coefficient of determination (R^2^), mean squared error (MSE), and Pearson’s correlation coefficient (r). The best model found was the XGBoost-R model [54]. Figure 12c shows a comparison of feature importances, revealing that the most important synthesis parameter was the volume of ethylenediamine. Such a result is understandable from an experimental point of view, as ethylenediamine acts as an alkaline catalyst and, as well, a reactant, which can improve the reaction, provide N-doping, and reduce defects in the prepared CDs: all of this may contribute to the higher quantum yields of CDs [54]. The trained ML algorithm allowed also for the optimization of the synthesis process.

**Figure 13:**
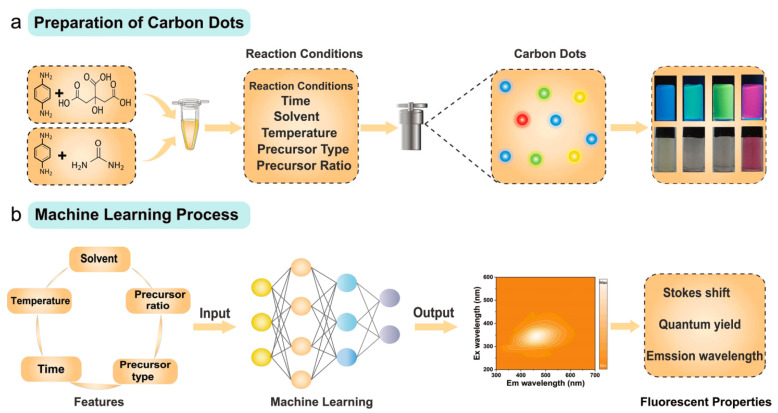
Scheme of performed machine learning modeling of multicolor CD synthesis workflow [55]. (**a**) Preparation of multicolor CDs to establish training datasets for machine learning. (**b**) Exploring the relationship between the synthesis parameters and the luminescence properties of CDs and predicting the photoluminescence properties of multicolor CDs using a machine learning model. Reproduced with permission from ref. [55].

Yet another study on the application of machine learning methods for the elucidation of the influence of synthesis parameters on the photoluminescence properties of carbon dots was conducted by Chen et al. [55]. Figure 13 shows a scheme of the conducted study. In the ML model, the input features consisted of the following reaction conditions: time of synthesis (4, 8, or 12 h), solvent used (anhydrous ethanol, water, or N,N-dimethylformamide), temperature of synthesis (140, 170, or 200 °C), precursor type (p-phenylenediamine and citric acid, or p-phenylenediamine and urea), and precursor ratio [55]. The output variables were Stokes shift, quantum yield, and emission wavelength [55]. The authors collected data for 270 syntheses in total. For each synthesized CD, a 3D fluorescence spectrum and the quantum yield were determined. The dataset was divided into training and validation datasets with a ratio of 9:1 [55]. The solvent input cannot be directly used in a computer, so Chen and cooperators chose five solvent properties (dielectric constant, density, specific heat capacity, viscosity, and boiling point) to represent the solvent characteristics [55]. The analysis based on Pearson’s correlation coefficient revealed that these five features were mathematically correlated, so the authors decided to transform them using principal component analysis (PCA). In this way, two principal components were obtained that accounted for 100% of the eigenvalues [55]. The authors compared the following six machine learning algorithms: extreme gradient boosting (XGBoost), random forest (RF), light gradient-boosting machines (LGBMs), ridge regression (Ridge), least absolute shrinkage and selection operator (LASSO), and support vector regression (SVR). The choice of the best algorithm was based on the R^2^ score and mean absolute error, and it turned out that the best machine was the random forest algorithm. Next, the rank importance algorithm was used to calculate the effect of the synthesis parameters on the photoluminescence properties [55].

Hong and cooperators utilized the extreme gradient boost regressor for machine learning of the photoluminescence intensity and emission centers of carbon dots synthesized starting from p-benzoquinone (PBQ) and ethylenediamine (EDA) in different solvents at room temperature [56]. The trained machine learning model was then used for the determination of the conditions of synthesis of various green and blue fluorescent CDs, which were subsequently applied for Fe^3+^ detection, sustained drug release, whole-cell imaging and poly(vinyl alcohol) (PVA) film preparation [56]. The authors collected data for four hundred different CDs prepared at different synthesis conditions. The mass of p-benzoquinone, volume of ethylenediamine, reaction time, and solvent used were the parameters that were varied [56]. Some basic analysis indicated that an increasing reaction time can lead to an increase in the maximal photoluminescence intensity and that ethanol as the solvent promotes greater photoluminescence intensity [56]. However, to elucidate clearly the relation between the fluorescence intensity of CDs and their synthesis conditions, machine learning was applied [56]. The trained ML model would also help in choosing the optimal reaction conditions. Similarly, as in the study described previously (ref. [55]), the input features for the solvent were (among others) the dielectric constant, viscosity, and density. Due to the correlation between some of the solvent parameters, determined using Pearson’s correlation analysis, the authors also used principal component analysis (PCA) to further reduce the dimensionality of the problem [56]. From six initial features of the solvent, two principal components were created that maintained 98.72% of the original information [56]. The regression models applied by the authors were the following: K-nearest neighbors, decision trees, random forest, support vector machines, convolutional neural networks, and XGBoost. The dataset was randomly shuffled and subsequently divided into the training and test sets at a ratio of 8:2 [56]. The grid search was used to optimize each machine learning model. The performances of different ML models were evaluated based on the root-mean-square error (RMSE), coefficient of determination (R^2^), and Pearson’s correlation coefficient (r). For such an evaluation, the best algorithm was XGBoost [56]. The authors additionally used the permutation importance algorithm to elucidate the influence of model features on the output variable. The permutation importance algorithm works with the following rule: the data corresponding to a certain feature, for which the importance is determined, are shuffled (other features remain unchanged); then, the ML algorithm is trained on new data, and the changes in the prediction metrics are determined. In the described study, the most important features were the volume of ethylenediamine and mass of the precursor [56]. The XGBoost model was also the most efficient (among the studied ML models) in the case of prediction of emission centers (i.e., the wavelengths of carbon dot emission peaks), and the analysis of feature importances showed the biggest influence being the mass of the precursor [56]. The trained XGBoost model also allowed for the synthesis of CDs with desired properties, e.g., useful in Fe^3+^ detection.

The last of the most recent studies on the application of machine learning techniques in the investigation of carbon dots is the study performed by Wang and cooperators [57]. They investigated the third-order nonlinear optical properties, which were tunable for a series of carbon dots prepared starting from oxalamide via a hydrothermal route [57]. The synthesized CDs exhibited a size-dependent effect on the third-order nonlinear refractive index and third-order nonlinear saturable absorption coefficient. The nonlinear optical properties of CDs were studied before; however, the possible factors that may influence such properties were not investigated clearly so far. They include the composition, structure, functional groups on the surface, size, and conjugation domain. The authors compared four distinct machine learning models implemented in the scikit-learn library: random forest, XGBoost, support vector machines, and gradient-boosting decision trees (GBDTs) [57]. The authors considered seven input features for ML describing the synthesis process: the reaction temperature, reaction time, reaction filling degree, mass of oxalamide, volume of concentrated sulfuric acid, heating rate, and type of water (pure, ultrapure, deionized, or distilled). The features were lowly correlated with each other [57]. The dataset consisted of 200 entries, from which 180 were used as the training set, and the rest were used as the test data. The best model was the GBDT, and it was further used to determine the feature importances. It was found that the most important synthesis parameter was the reaction time [57]. The second most important parameter was the reaction temperature, and the third was the reaction filling degree. The trained ML model allowed for speculations on the influence of the reaction time on the nonlinear optical properties of the synthesized CDs, for example, that when the reaction is less than 4 h, the nonlinear optical responses are negligible.

### 3.2. Machine Learning-Assisted Investigations of Metal Nanoparticles

The smaller amount, in comparison to the case of carbon dots, of the recent literature on applications of ML in studies of nanostructures concerns metal nanoparticles. The studies focused mainly on the synthesis process of metal nanoparticles, as well as on their properties such as structure, size, and toxicity.

**Figure 14:**
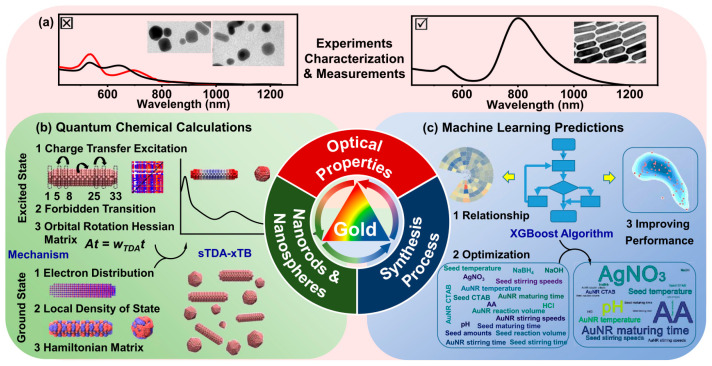
Summary of combined experimental, quantum chemical, and machine learning study of the synthesis of GNR-GNS heterodimers [58]. (**a**) Typical absorption spectra of GNR-GNSs (left chart) and high-quality pure GNRs (right). The decrease in the NIR absorption of GNRs by the GNSs may be observed. (**b**) Quantum chemical calculation, addressed from both ground and excited states, attributes the decrease in NIR absorption of the GNR-GNS system to all of these changes in the overall electronic structure and excitation behavior of a GNR affected by adjacent GNSs. (**c**) ML predictions not only associate the GNR performance with its synthesis process directly but also optimize the synthesis and further refine the optical properties of GNRs in validated experiments. Reproduced with permission from ref. [58].

Yin et al. combined quantum chemical calculations with the machine learning approach to investigate the synthesis of gold nanorods (GNRs) [58]. The understanding of the optical properties of GNRs in the colloidal state is very important from the point of view of their applications. However, in the synthesis process of GNRs, concomitant gold nanospheres (GNSs) are also formed, influencing the properties of the final product [58]. The authors studied the mechanism of this phenomenon by constructing heterodimeric nanoparticle consisting of GNR-GNS and performing quantum chemical calculations of their optical and electronic properties [58]. It turns out that the gold nanospheres inhibit certain charge-transfer excitations of adjacent gold nanorods [58]. The authors collected data for 310 GNR-GNS dimers synthesized as colloidal solutions with seed-mediated growth [58]. The approximate dimensions, based on the presented electron microscopy images, of the studied nanorods were 15–25 nm in diameter and several dozen nm in length, while the diameter of the nanospheres was in an approximate range of 10–100 nm. The absorption spectra were recorded for each heterodimer. In total, 11 machine learning models were compared. Among them, the XGBoost model was the most efficient, as it reached a prediction accuracy of over 94% [58]. Machine learning was further used to determine the influence of synthesis conditions on the optical properties of GNR-GNS colloids, avoiding complicated and time-consuming experimental work. The trained ML model also allowed for the optimization of the synthesis. Figure 14 shows a summary of the investigation performed by Yin and cooperators. Quantum calculations were performed using the extended tight-binding-based, simplified Tamm–Dancoff approximation (sTDA-xTB) to calculate the absorption spectra of excited-state GNRs coexisting with GNSs at a different spacing [58]. Based on these calculations, the authors determined that the NIR absorption spectra change significantly when a GNR-GNS dimer with 2.4 nm spacing is formed [58]. With the distance lowering from 72.4 nm to 2.4 nm, the peak position remains unchanged until the distance shrinks to 10 nm or lower; in this case, the peak started to red-shift [58]. To further investigate this observation, the authors studied HOMO and LUMO of the gold nanoparticles and found that the electronic properties of GNRs in the ground state soundly change when GNSs are close to them [58].

**Figure 15:**
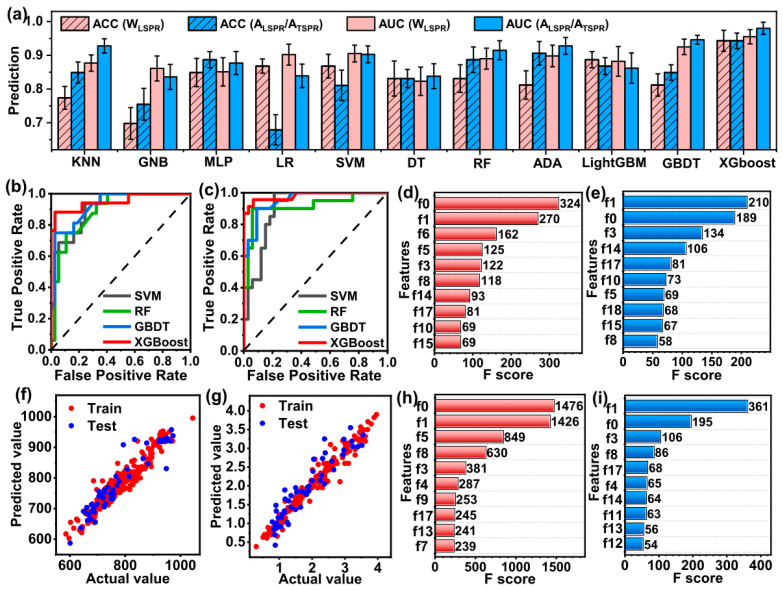
Comparison of different ML models used to study the optical properties of GNR-GNS colloids. (**a**) Prediction accuracy (ACC) and AUC values calculated using various ML models for the longitudinal surface plasmon resonance absorption peak wavelength (W_LSPR_) and the ratio of the maximum longitudinal absorbance versus transverse LSPR bands (A_LSPR_/A_TSPR_) of GNR-GNS solutions. (**b**) W_LSPR_ and (**c**) A_LSPR_/A_TSPR_. Importance scores of the top 10 processing features retrieved from the XGBoost classifier for predicting (**d**) W_LSPR_ and (**e**) A_LSPR_/A_TSPR_. A scatter plot showing regression correlations between the experimental and predicted values by the XGBoost regressor: coefficients of determination (R^2^) of train and test data are (**f**) 0.92 and 0.89 for predicting W_LSPR_ and (**g**) 0.96 and 0.86 for predicting A_LSPR_/A_TSPR_, respectively. Importance scores of features retrieved from the XGBoost regressor for predicting (**h**) W_LSPR_ and (**i**) A_LSPR_/A_TSPR_. Reproduced with permission from ref. [58].

For the machine learning study, Yin et al. used 11 classifiers: K-nearest neighbors, Gaussian Naive Bayesian (GNB), multilayer perceptron (MLP), logistic regression (LR), support vector machines, decision trees, random forest, AdaBoost (ADA), gradient-boosting decision trees (GBDTs), light gradient-boosting machines (LightGBMs), and extreme gradient boosting (XGBoost) [58]. The dataset had 19 input features. The following metrics were used for the evaluation of the models: accuracy, precision, recall, receiver operating characteristic (ROC), and area under the ROC curve (AUC) [58]. The best was the XGBoost model, with a prediction accuracy greater than 94% and AUC up to 98% [58]. Figure 15 presents a detailed comparison of the studied machine learning algorithms. The XGBoost algorithm was further used to determine the feature importances, revealing that the most important factors in the GNR preparation were AgNO_3_ and ascorbic acid [58].

**Figure 16:**
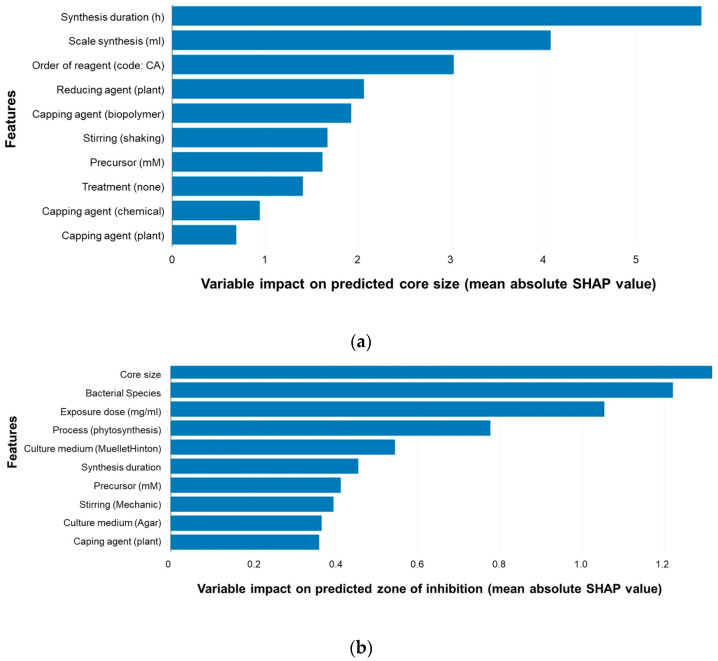
SHAP values for the prediction of the (**a**) core size of Ag NPs and (**b**) antibacterial activity of Ag NPs. Reproduced under the Creative Common license from ref. [59].

The control of the physical and chemical properties of silver nanoparticles is very important for their applications and toxicity; thus, Furxhi et al. used machine learning to determine the influence of conditions of Ag NP synthesis on their properties [59]. The authors collected literature data (219 studies), including parameters like experimental conditions, reactant concentrations, physicochemical properties, antibacterial activities, and toxicological profiles [59]. The data were collected manually by a team of researchers with prior expertise in the synthesis and characterization of nanomaterials. The following experimental conditions may influence the synthesized Ag NPs and, thus, were included in the study: the reagent concentrations (silver precursor and reducing agent), pH of the reaction mixture, reaction temperature, stabilizing agents (surfactants and polymers), reaction time, use of an external field (electric, magnetic, and ultrasound), and use of biological materials (natural reducing agents and stabilizers). Furthermore, the properties of synthesized Ag NPs (such as dispersibility, solubility, and hydrophobicity) may be tuned via the functionalization of nanoparticles with different coatings, which, on the other hand, influence biodurability and bioaccessibility [59]. As may be seen, the problem is multidimensional and relatively complex, which justifies the usage of machine learning techniques. The authors used one-hot encoding for the categorical variables and the PyCaret regression library as a source of machine learning algorithms [59]. This library contains more than 18 regression models, for example, linear regression, decision trees, support vector machines, elastic net, huber, ridge, etc. [59]. The models were validated using 10-fold cross-validation (in general, cross-validation improves the generalization of the model and its performance on unseen data). The authors used the following validation metrics: root-mean-square error (RMSE), mean absolute error (MAE), and R-squared. The features’ impact on the corresponding target variables was estimated using SHAP (Shapley Additive exPlanations) average values, and the most significant parameters were features like the synthesis time, scale of synthesis, and type of capping agent [59,60,61,62]. Figure 16 shows the SHAP values when the target variables were the core size of Ag NPs and the zone of inhibition (depending on antibacterial activity) [59].

**Figure 17:**
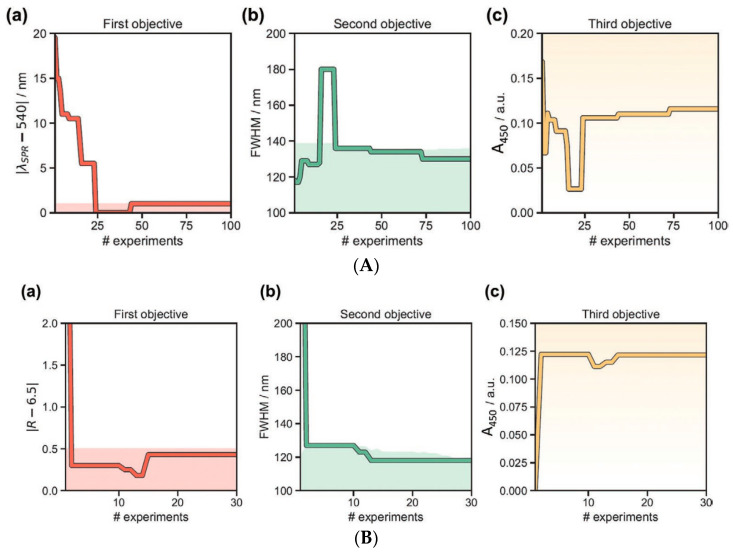
Variation in targeted AuNP characteristics of the current best recipe found during the optimization of the synthesis of (**A**) large AuNPs and (**B**) small AuNPs. Reproduced with permission from ref. [63].

The study performed by Tao et al., which combined microfluidics and machine learning, concerned the synthesis of gold nanoparticles [63]. Such a combination offers accelerated studies of reaction conditions for nanoparticles synthesis. The authors constructed an autonomous machine learning-driven oscillatory microfluidic platform for the synthesis of metal nanoparticles [63]. The platform was capable of conducting syntheses under different experimental conditions and reaction times, with varied NP dimensions. At the same time, the platform was able to conduct spectroscopic characterization of NPs, perform ML analysis of the spectroscopic properties of the synthesized NPs, and identify the reaction conditions required to achieve NPs with the desired optical properties [63]. The authors used the Gryffin machine learning algorithm, which utilizes Chimera, which is a new approach to multi-objective optimization, allowing for the ranking of multiple target variables in a decreasing order of importance [63,64]. The algorithm was able to perform online optimization of the synthesis of large and small Au NPs as well (see Figure 17) [63].

The authors also used the machine learning approach to elucidate the correlations between reaction conditions and Au nanoparticle properties [63]. They performed, in total, 160 experiments and applied three distinct ML algorithms—random forest, Gaussian process, and support vector regression—to check if the variation in reaction conditions (i.e., concentration of HAuCl_4_, concentration of PVP, concentration of D-glucose, concentration of NaOH, and the reaction time) is linked with the variation in spectroscopic properties of synthesized AuNPs [63]. The SVR model had a higher prediction accuracy than the rest of models; thus, it was used to perform the interpretation of the model. For this purpose, the Shapley additive explanations (SHAP) algorithm was used [63]. Based on this approach, it was found that a higher concentration of PVP leads to the formation of Au nanoparticles with a smaller size; such a conclusion was consistent with other studies [63]. Another conclusion drawn from the SHAP analysis was that the achievement of a high yield of AuNPs requires higher concentrations of D-glucose, HAuCl_4_, and NaOH, while the concentration of PVP and the reaction time are less important in this case [63].

**Figure 18:**
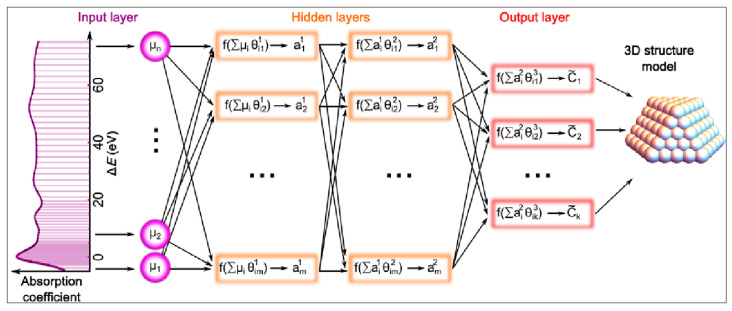
Schematic representation of artificial neural network-based method for prediction of nanoparticle size and shape. Reproduced with permission from ref. [65].

Timoshenko and cooperators presented an interesting machine learning-based study of the three-dimensional structure of metallic NPs [65]. As metallic nanoparticles often find applications in the field of heterogeneous catalysis, their shape may influence the catalytic activity; thus, the determination of this is an important issue. The authors utilized X-ray absorption near-edge structure (XANES) spectroscopy and supervised machine learning for refining the 3D structure of metal NPs [65]. The ML algorithm was trained on XANES spectra calculated using ab initio methods. In the study, it was demonstrated that it is possible to determine the average size, shape, and morphology of platinum nanoparticles based on their experimental XANES spectra [65]. Figure 18 presents a schematic representation of the performed machine learning modeling.

## 4. Conclusions

This review provides knowledge of recent studies describing analyses of a wide range of factors on quantum dot properties. While experimental investigation of the influence of some factors on quantum dots can be problematic and sometimes impossible, theoretical calculations become a powerful tool for studies of nanocrystals, which helps to explore the nature of changes in charge transport, electron–hole recombination, energy relaxation, electron–phonon coupling, the lifetime of excited states, light absorption, and photoluminescence. Moreover quantum mechanical and machine learning simulations have a wide range of use in practical applications, providing the essential data for designing adsorbents, catalytic processes, as well as optoelectronic and photovoltaic devices.

## Figures and Tables

**Table 1:** Summary of presented DFT studies: investigated systems and main conclusions.

Studied System	Conclusions	Reference
CsPbBr_x_I_y_	Increase in iodine content leads to longer electron–hole recombination time.	[16]
MAPbI_3_ QDs/TiO_2_	Electron delocalization decreases in the series Sn doped > MAPbI_3_/TiO_2_ > Br doped > Cl doped.	[18]
CdSe	Deep hole traps decrease but do not eliminate Auger recombination. Combination of deep and shallow hole traps enhances trapping but, at the same time, opens new Auger channels.	[19]
PbS	Oxidation of n-doped and reduction of p-doped NCs causes formation of traps.	[20]
	Molecular oxygen adsorption by PbS causes formation of in-gap states.	[24]
	Stokes shift in polydisperse PbS solid film is higher than isolated quantum dots. Main effect causing excessive FC shifts in PbS is some electronic defects.	[25]
CdSe, PbS, and CsPbI_3_	Z-type ligands strongly influence CdSe more than PbS. CsPbI_3_ is resistant to Z-type ligands but prone to oxidation.	[21]
Functionalized triangular and hexagonal GQDs	Large band gap appears in the case of armchair–hexagonal, armchair–triangular, and zigzag–hexagonal functionalized GQDs, while zigzag–triangular quantum dots have a small energy gap.	[22]
GQD/TiO_2_ composites	Donor–acceptor coupling is mainly caused by π-electron interactions and leads to adiabatic ET.	[23]
Heteroatom-doped GQDs	In all cases, doping caused a decrease in absorption, nitrogen and sulfur doping caused a red-shift, and boron and phosphorus exhibited a blue-shift. Unoxidized boron doping causes a significant decrease in absorption. Oxygen-containing group surface modification causes a decrease in absorption and a red-shift.	[26,27,28]
	The doping of graphene increases binding energy with water, carbon monoxide, and ammonia molecules.	[30]
	The most negative adsorption energy value of 5-fluorouracil is −47.29 kcal/mol in the case of aluminum nitrogen doping.	[31]
Cu_2_O-decorated SnO_2_ nanostructure	The studied nanostructure is potentially a good creatinine sensor. The structure of adsorbed molecule and binding energy of creatinine and other particles were studied. The obtained absolute values of binding energies grow in the series creatinine > NHS > uric acid > glucose > urea > L-cysteine > dopamine > ascorbic acid > cholesterol.	[29]
Functionalized 2D AlN	The adsorption strength grows in the following order: AlN-QDs-edg-2CO-formalin < AlN-QDs-edg-2CO-methanol < AlN-QDs-edg-2CO-ethanol < AlN-QDs-OH-formalin < AlN-QDs-OH-methanol < AlN-QDs-OH-ethanol.	[32]
Cs_2_CuBr_4_ and CsPbBr_4_	Cs_2_PbBr_4_ in comparison to CsPbBr_3_ led to d-band closer to Fermi level, causing better CO_2_ adsorption, decrease in activation energy of slowest step, and change in route to formation of CH_4_ instead of CO.	[33]
ZnO/RGO, CoO/RGO, and SnO/RGO	Rate-limiting step in electrocatalytic reduction of nitrogen is hydrogenation of adsorbed nitrogen to *N_2_H. NH_3_ yield growth occurs in the series ZnO/RGO < CoO/RGO < SnO/RGO, while FE growth occurs in the series ZnO/RGO < SnO/RGO < CoO/RGO.	[32,33,34]
Carbon QD-decorated g-C_3_N_4_	Carbon dots form type-II Van der Waals heterojunction with g-C_3_N_4_, which can be utilized in water-splitting process.	[37]
MoS_2_	Edge defects cause an increase in the catalytic performance of oxygen evaluation. The limiting step for this process is the reaction between OH^−^ and adsorbed O atom. During the electrochemical process with external potential of 0.6 V and pH = 14, the limiting step is O_2_ desorption.	[38]
BiSbO_4_ QDs and BiSbO_4_ QDs/carbon dots	The photocatalytic performance of BiSbO_4_ QDs on carbon dots (CDs) composite is better than pristine BiSbO_4_ QDs because carbon atoms form an electron depletion layer on the inner side of BiSbO_4_.	[39]
